# Epidemiology of Patients with Head Injury at a Tertiary Hospital in Rwanda

**DOI:** 10.5811/westjem.2021.4.50961

**Published:** 2021-11-05

**Authors:** Naz Karim, Lise Mumporeze, Vizir J.P. Nsengimana, Ashley Gray, Alexis Kearney, Adam R. Aluisio, Zeta Mutabazi, Janette Baird, Camille M. Clancy, Derek Lubetkin, Jean Eric Uwitonze, Jeanne D’Arc Nyinawankusi, Menelas Nkeshimana, Jean Claude Byiringiro, Adam C. Levine

**Affiliations:** * Warren Alpert School of Medicine, Brown University, Department of Emergency Medicine, Providence, Rhode Island, United States of America; † University of Rwanda, College of Medicine and Health Sciences, Department of Anesthesia, Critical Care, and Emergency Medicine, Kigali, Rwanda; ‡ University Teaching Hospital-Kigali (UTH-K), Department of Accident & Emergency Medicine, Kigali, Rwanda; § Service d’Aide Médicale Urgente (SAMU), Rwanda Ministry of Health, Kigali Rwanda; * University Teaching Hospital-Kigali (UTH-K), Department of Accident & Emergency Medicine, Kigali, Rwanda; † University Teaching Hospital-Kigali (UTH-K), Division of Clinical Education and Research, Kigali, Rwanda; ‡ Warren Alpert School of Medicine, Brown University, Department of Emergency Medicine, Providence, Rhode Island, United States of America

## Abstract

**Introduction:**

Traumatic injuries disproportionately affect populations in low and middle-income countries (LMIC) where head injuries predominate. The Rwandan Ministry of Health (MOH) has dramatically improved access to emergency services by rebuilding its health infrastructure. The MOH has strengthened the nation’s acute emergency response by renovating emergency departments (ED), developing the field of emergency medicine as a specialty, and establishing a prehospital care service: Service d’Aide Medicale Urgente (SAMU). Despite the prevalence of traumatic injury in LMIC and the evolving emergency service in Rwanda, data regarding head trauma epidemiology is lacking.

**Methods:**

We conducted this retrospective cohort study at the University Teaching Hospital of Kigali (UTH-K) and used a linked prehospital database to investigate the demographics, mechanism, and degree of acute medical interventions amongst prehospital patients with head injury.

**Results:**

Of the 2,426 patients transported by SAMU during the study period, 1,669 were found to have traumatic injuries. Data from 945 prehospital patients were accrued, with 534 (56.5%) of these patients diagnosed with a head injury. The median age was 30 years, with most patients being male (80.3%). Motor vehicle collisions accounted for almost 78% of all head injuries. One in six head injuries were due to a pedestrian struck by a vehicle. Emergency department interventions included intubations (6.7%), intravenous fluids (2.4%), and oxygen administration (4.9%). Alcohol use was not evaluated or could not be confirmed in 81.3% of head injury cases. The median length of stay (LOS) in the ED was two days (interquartile range: 1,3). A total of 184 patients were admitted, with 13% requiring craniotomies; their median in-hospital care duration was 13 days.

**Conclusion:**

In this cohort of Rwandan trauma patients, head injury was most prevalent amongst males and pedestrians. Alcohol use was not evaluated in the majority of patients. These traumatic patterns were predominantly due to road traffic injury, suggesting that interventions addressing the prevention of this mechanism, and treatment of head injury, may be beneficial in the Rwandan setting.

## INTRODUCTION

Traumatic injury is a major public health problem leading to approximately five million deaths worldwide.^1^,^2^ Approximately 85% of the world population lives in low and middle-income countries (LMIC),^3^ where 90% of injury occurs.^4^ Morbidity and mortality due to head and spinal injuries predominate in LMICs.^3^,^5^–^9^ The World Health Organization (WHO) identifies motor vehicle collisions (MVC) as the most common cause of head injury. The WHO estimated an 80% rise in the total number of traffic deaths by 2020.^7^,^10^ Several studies have emphasized the importance of improving access to emergency health services, which could reduce morbidity and mortality specifically in LMICs. 11–21 An estimated 45% of deaths and 36% of disability-adjusted life years in LMICs could be addressed by the implementation of emergency care systems. 3,13

The Rwandan Ministry of Health (MOH) has made dramatic improvements in expanding access to healthcare, rebuilding its health infrastructure by establishing emergency medicine (EM) as a specialty, developing an EM residency program, and creating a prehospital care service.^22^ In 2007, the MOH implemented an emergency ambulance system, Service d’Aide Medicale Urgente (SAMU), to provide greater healthcare access.^22^ This was a critical step towards improving trauma response, as correcting hypoxia and hypotension during prehospital care has been shown to improve outcomes.^23^,^24^

Despite the prevalence of traumatic injury in LMICs, and the evolving emergency service in Rwanda, data regarding head trauma epidemiology is lacking. Defining the epidemiology of head injury in Rwanda may lead to more focused preventative and medical management strategies that impact disability and mortality rates for years to come.^3^,^5^–^6^ This study evaluates the distribution and outcomes of patients with head injury transported by SAMU to University Teaching Hospital-Kigali (UTH-K) in Kigali, Rwanda.

## METHODS

### Study Setting

We conducted a retrospective cohort study at UTH-K. The primary referral and training hospital in Rwanda, UTH-K is a 550-bed facility located in the city of Kigali amongst a population of 1.1 million people.^25^ The hospital’s emergency department (ED) is responsible for the acute management of all adult patients, as well as pediatric and obstetric patients with traumatic injuries.

### Data Collection

Trained research assistants (RA) linked prehospital, patient run sheets to ED health records. The linkage was performed through queries of the hospital electronic billing system, named Open Clinic. A composite patient identification index based on patient name, date of birth, date of service, and address in Open Clinic were matched within the SAMU database to confirm identity. Data extracted from these records included initial vital signs, Glasgow Coma Scale, administration of glucose and fluids, diagnosis of head injury, hospital length of stay (LOS), and condition upon discharge. The extracted data was de-identified and entered into a secured database Researh Electronic Data Capture (REDCap Consortium, Vanderbilt University, Nashville, TN) for statistical analysis. The study was approved by the Lifespan Institutional Review Board, Rwanda National Ethics Committee (RNEC), and the UTH-K Research Committee.

**Population Health Research Capsule**

What do we already know about this issue?

*This is the first study to assess the epidemiology of head injury in patients receiving prehospital care followed by emergency care in Rwanda.*

What was the research question?

*We investigated the demographics, mechanism, and degree of acute medical interventions amongst prehospital patients with head injury presenting to University Hospital-Kigali.*

What was the major finding of the study?

*Our data showed that 56.5% of patients with traumatic injuries were diagnosed with head injury, and 78% were the result of motor vehicle collisions.*

How does this improve population health?

*Defining the epidemiology of head injury in Rwanda may lead to more focused preventative and medical management strategies that impact disability and mortality rate.*

### Population

Selection criteria included all patients transported to the ED at UTH-K by SAMU for traumatic head injury from December 2012–February 2015. Head injury was defined as patients with a chief complaint of head injury, craniofacial trauma on physical examination, or computed tomography (CT) reports of cranial trauma. Patients transported by SAMU with non-traumatic ailments or injury other than head trauma were ineligible for the study. We excluded patients if their records could not be linked to the ED.

### Statistical Analysis

We conducted statistical analysis using Stata version 14.0 (StataCorp LLC, College Station, TX). Measures used to describe the distribution of data included mean, median, and interquartile ranges (IQR).

## RESULTS

**Figure 1:**
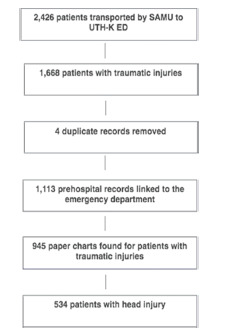
Flow diagram of patient enrollment.

During the study period, 2,426 patients were transported by SAMU, of whom 1,669 were found to have traumatic injuries. Prehospital data was linked to emergency health records, and data was successfully abstracted from 945 (56.6%) cases. Amongst the 945 patients with traumatic injuries, 534 (56.5%) were diagnosed with a head injury (Figure 1).

**Figure 2:**
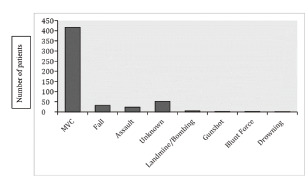
Mechanism of injury in patients with head injuries. *MVC*, motor vehicle collision.

The cohort was composed of 429 males (80.3%) and 105 (19.7%) females. The median age was 30 (IQR: 25–36). Almost 78% (n = 417) of head injuries occurred due to MVCs (Figure 2). The type of accidents included the following: 15.3% (n = 64) vehicle-motorcycle; 11.3% (n = 47) motorcycle-pedestrian; 10.0% (n = 42) vehicle-pedestrian; 8.3% (n = 35) single vehicle only; 7.7% (n = 32) single motorcycle only; 4.6% (n = 19) motorcycle-motorcycle; 4.1% (n = 17) motorcycle-unknown; 4.0% (n = 17) vehicle-vehicle; 1.4% (n = 6) vehicle-bicycle; 1.4% (n = 6) motorcycle-bicycle; and 31.4% (n = 131) accident types were unknown, unreported, or missing data regarding the type of accident (Figure 3).

Of patients with a head injury in the ED, 431 (80.7%) had documented vital signs, 14 (2.6%) patients were hypotensive, of whom 13 received intravenous (IV) fluids, and 30 (5.6%) were hypoxemic, of whom 26 received oxygen. Glasgow Coma Score (GCS) was recorded for 416 (79.9%) patients with head injuries. There were 32 (6.0%) patients with a head injury and a documented GCS of less than eight, 14 of whom were subsequently intubated in the ED for this indication. One patient could not be successfully intubated, one person was intubated in the prehospital setting, and 16 patients had no further documentation. There were approximately 17% (n = 93) confirmed cases of alcohol use amongst patients with head injuries. In 81.3% (n = 434) of cases, alcohol use was not evaluated or could not be confirmed.

The median LOS in the ED was two days (IQR: 1–3). The overall mortality prevalence was 7.3%, with 4.1% of deaths occurring in the ED. Seventeen (9.2%) admitted patients with a head injury died. A total of 184 patients were admitted to the hospital. Craniotomies were performed on 24 patients while hospitalized. The median hospital LOS was 13 days (IQR: 7–25).

## DISCUSSION

The WHO has identified MVCs as the most common cause of head injury.^7^,^10^ The goal of this study was to understand the epidemiology of head injury amongst prehospital patients in Rwanda. In this retrospective analysis, head injury was prevalent amongst patients with traumatic injuries who were transferred by prehospital providers to UTH-K, with the majority of head injuries attributed to MVCs.

Public health strategies focused on road safety are critical to the prevention of head injuries. Recently Rwanda has focused its efforts on improving road safety. In 1996, the World Bank situation report measured one traffic accident every 2.5 hours in Rwanda.^26^ A campaign of reforming infrastructure and safety began, and by 2001 regulations were published requiring seat belts, speed limits, vehicle inspections, and blood-alcohol levels.^26^ In 2003 further regulation regarding helmet use for motorcycles was published. As a result, Rwanda saw the death rates drop 30%; and in 2006, Rwanda was recognized by the WHO for its efforts.^24^ Nevertheless, our data set suggests that there is still work to be done.

Head injury was overall most prevalent amongst males in this study. The majority of injuries occurred due to motorcycle collisions with vehicles, but one in six head injuries involved a pedestrian struck by a vehicle. More importantly, alcohol use was unknown in the majority of cases involving head injuries (81%). The data was collected either by history or not documented. The 2004 WHO global health risks report attributes 20% deaths in MVCs to alcohol use.^27^ Given the known association of alcohol use and morbidity further research should be prioritized to determine the link specifically to head trauma.

Of the head injury patients, the prevalence of hypoxia was greater than the prevalence of hypotension. In general, appropriate interventions were taken to manage these critically ill patients, such as supplementing oxygen or providing bolus infusions. Notably, however, 50% of patients with GCS less than 8 were not intubated. Further research is required to determine the reason for the lack of intubation in patients with a head injury, which may include the continual need for intubating supplies, equipment, and sedative medications.

## LIMITATIONS

Patients who were very ill and unable to identify themselves were transferred to the ED and listed as “inconnu” in French, or “unknown.” While we included these unknown patients in the study, they could not be linked to the ED records due to a lack of demographic variables. As a result, recorded GCS scores may be high for head injury patients because unlinked patients were not included. It is likely that these critical patients would have the lowest GCS scores. Another limitation involves data abstraction solely from a prehospital cohort. It is, therefore, difficult to locate patients with traumatic injuries who arrived at the ED by other means of transportation. Another limitation involves data abstraction. Approximately 57% of charts were abstracted given the limitation of linking health records. The retrospective design in an LMIC resulted in a significant number of missing data points as well. Although a prospective study was not possible due to resource limitations, the data abstraction was similar to prospective studies in the region.^28^–^31^

The patients who were not linked may have been demographically different with varying injury patterns. Thus, data cannot be extrapolated to all patients with traumatic injuries in Rwanda. Additionally, the high rate of unknown mechanisms of trauma may be related to lack of adequate documentation; further quality-related studies are needed to assess this issue thoroughly. Documentation is incredibly important; unfortunately, it is missing quite often in LMICs. We hope to bring this issue to light so that it may allow for quality improvement studies in the future. Finally, overall outcomes regarding physical, cognitive, and psychological functioning following trauma could not be analyzed given that such information is not yet available for abstraction from the health records of patients in the ED or those admitted to the hospital. Data was also lacking regarding transfers to other hospitals in Rwanda, patients who were discharged, and patients lost to follow-up.

## CONCLUSION

A significant proportion of prehospital patients with trauma have a head injury. This study found that such injury is prevalent amongst males and pedestrians. There is a need to document the diagnosis and documentation of alcohol use as future studies may identify this cause as an additional risk factor for head injury. The epidemiologic description of patients with traumatic head injuries can provide critical information to help guide strategies in the prevention, treatment, and management of traumatic head injuries in Rwanda. Long-term injury surveillance and geospatial mapping should be considered in the future to provide additional information about the distribution of head injuries. Further data analysis is required to analyze the association of head injuries on patient outcomes. A prospective study involving all hospitals may better define the epidemiology of head injury in Rwanda.
